# Soil properties in agricultural systems affect microbial genomic traits

**DOI:** 10.1093/femsmc/xtaf008

**Published:** 2025-06-24

**Authors:** Tim Goodall, Susheel Bhanu Busi, Robert I Griffiths, Briony Jones, Richard F Pywell, Andrew Richards, Marek Nowakowski, Daniel S Read

**Affiliations:** UK Centre for Ecology & Hydrology (UKCEH), Wallingford, OX10 8BB, United Kingdom; UK Centre for Ecology & Hydrology (UKCEH), Wallingford, OX10 8BB, United Kingdom; Bangor University, School of Environmental and Natural Sciences, Gwynedd, LL57 2DG, United Kingdom; UK Centre for Ecology & Hydrology (UKCEH), Gwynedd, LL57 2UW, United Kingdom; UK Centre for Ecology & Hydrology (UKCEH), Wallingford, OX10 8BB, United Kingdom; Agrii - Masstock Arable (UK) Limited, Andoversford Link, Andoversford, Cheltenham, GL54 4LZ, United Kingdom; Wildlife Farming Services Limited, Chesterwood, Chesterton, Bicester, OX261UN, United Kingdom; UK Centre for Ecology & Hydrology (UKCEH), Wallingford, OX10 8BB, United Kingdom

**Keywords:** genomic traits, metabarcoding, land use, soil properties, agricultural systems, microbial ecology, social niche breadth

## Abstract

Understanding the relationships between bacteria, their ecological and genomic traits, and their environment is important to elucidate microbial community dynamics and their roles in ecosystem functioning. Here, we examined the relationships between soil properties and bacterial traits within highly managed agricultural soil systems subjected to arable crop rotations or management as permanent grass. We assessed the bacterial communities using metabarcoding and assigned each amplicon trait scores for rRNA copy number, genome size, and guanine-cytosine (GC) content, which are classically associated with potential growth rates and specialization. We also calculated the niche breadth trait of each amplicon as a measure of social ubiquity within the examined samples. Within this soil system, we demonstrated that pH was the primary driver of bacterial traits. The weighted mean trait scores of the samples revealed that bacterial communities associated with soils at lower pH (<7) tended to have larger genomes (potential plasticity), have more rRNA (higher growth rate potential), and are more ubiquitous (have less niche specialization) than the bacterial communities from higher pH soils. Our findings highlight not only the association between pH and bacterial community composition but also the importance of pH in driving community functionality by directly influencing genomic and niche traits.

## Teaser

Bacterial genomic traits are related to soil properties in agricultural systems.

## Introduction

Microbial genomic traits such as genome size and rRNA copy number are key indicators of niche breadth and ecological strategies in soil bacterial communities (von Meijenfeldt et al. 2023). These traits reflect the ability of microorganisms to adapt to varying environmental conditions (Chuckran et al. 2021), including nutrient availability, pH, and organic matter (OM) content. Understanding the relationship between soil properties and microbial genomic traits is crucial for advancing our knowledge of microbial ecology, particularly in agricultural systems, where soil management practices can significantly alter these properties.

Agricultural soils represent a significant portion of land use in the United Kingdom, with 70% of the total land area classified as Utilised Agricultural Area (UAA) (DEFRA. 2023). In 2023, the total arable area in the UK was reported to be just over 6.0 million hectares, accounting for ~36% of the UAA (DEFRA. 2023). Soils in these agricultural systems vary widely in pH and OM content, which are critical factors that influence soil dwelling microbial communities and their genomic traits (Zhang et al. 2020, Naylor et al. 2022). Previous studies have shown that conversion to arable land leads to modified microbial communities, where the consequences include reductions in functionality and decreases in genes relating to important biogeochemical cycles, including those that influence the cycling and fate of carbon, phosphorous and nitrogen containing compounds (Peng et al. 2024). Simultaneously, the age of managed and permanent grass fields, key for providing food and income, affects the microbial community, especially bacteria, through the influence of soil physicochemical properties, such as pH (Seaton et al. 2022).

**Figure 1.**
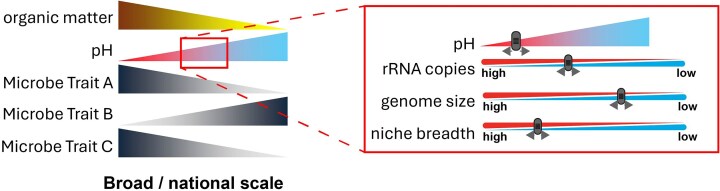
Conceptual diagram (hypothesis)—at broad scale, the environmental variables soil OM and soil pH are coupled. Here we examine highly managed soils where OM and pH are decoupled and examine the relationship of these key environmental variables upon the microbial traits rRNA copies (growth rate potential), genome size (metabolic versatility), and niche breadth (ubiquity).

Microbial metabolic versatility (Klappenbach et al. 2000) and the ability of taxa to thrive in diverse and fluctuating environments (Wang et al. 2023) are typically associated with larger genome sizes and increased rRNA copy numbers. Conversely, smaller genomes and fewer rRNA copies suggest specialization and efficiency under more stable or resource-limited conditions (Chuckran et al. 2021). The ability of soil microbial communities to adapt to changes in soil properties, such as pH and OM content, is reflected in these genomic traits, which in turn influence the overall health and functionality of the soil ecosystem. For example, Wilhelm et al. (2023) demonstrated a positive relationship between genome size and rRNA copy number in soil bacteria. These genomic traits are relevant for understanding the classical concept of ecological niche breadth, which defines the range of conditions under which organisms can survive in Carscadden et al. (2020). Although niche breadth has previously been assessed with respect to environmental variables, it has been limited to specific taxa (Bennett and Lenski 1993) or environments (Kuang et al. 2012). Importantly, the notion of generalists, taxa found in many samples or predefined habitats, and specialists, taxa that are rare or highly fastidious (Cobo-Simón and Tamames 2017), have not been assessed in soils under land use effects, necessitating the need to understand how anthropogenic influences affect the social niche breadth (SNB) of microorganisms. We hypothesised that the environmental factors pH and OM play a significant role in shaping microbial communities and their genomic traits and tested their relationships within highly managed agricultural soils where the broadly observed inverse relationship between pH and OM is decoupled. Specifically, we propose that gradients in pH and OM differentially influence individual microbial traits including ubiquity and genome size (Fig. 1). To test the relationship between microbial traits and these environmental influences, we assessed microbial communities and their traits in soils from arable and grassland sites across 245 samples from 69 fields at 20 farms in southern United Kingdom/England. By leveraging systematic sampling and molecular methods, our study provides novel insights into the relationship between soil properties and microbial genomic traits in agricultural systems, especially arable and permanent (≥4 years) grass fields, the latter of which are relatively unknown. Our findings highlight the importance of soil pH as a key factor influencing microbial genomic traits, such as size and niche breadth, challenging the view that nutrient availability (carbon content) determines these traits in isolation. These results have important implications for understanding microbial adaptation in agricultural soils and the management of soils in these systems for specific ecological outcomes.

## Methods

### Sample collection

During the Autumn of 2019, 245 subsurface (<15 cm) soils were sampled from 68 fields in southern England, by Agrii-Masstock Arable (UK) Limited. Of these 214 samples were from fields designated as arable, having been in cereal (wheat and barley) and oil seed rape rotation for ≥4 years; 15 fields were mixed arable and grass rotations of barley and grass for ≥4 years; and 16 fields were permanent grass, where no other crop had been grown for ≥4 years (Supplementary Table 1). The samples were collected into sample boxes and transported immediately to NRM—Cawood Scientific (Berkshire, UK) for processing.

### Physicochemical measurements and DNA extraction

Soil physicochemical measurements were made at NRM—Cawood Scientific (Berkshire, UK): pH, OM content as a percentage of dry mass by loss on ignition (LOI); phosphorus, potassium, magnesium as mg per litre; and sand (0.063–2.000 mm), silt (0.002–0.063 mm), and clay (<0.002 mm) content as a percentage of total. Sample properties are listed in Supplementary Table 1.

DNA extraction was performed on 0.2 g of dried, milled soil sample using the Qiagen Power Soil HTP-96 kit (Cat. No. 12955-4) following the manufacturer's instructions.

### PCR and sequencing

The extracted DNA was used to generate metabarcoding sequence data. Briefly, amplicons were produced using a two-step amplification approach, with Illumina Nextera tagged primers targeting the V4-5 region of 16S rRNA: 515f GTGYCAGCMGCCGCGGTAA and 806r GGACTACNVGGGTWTCTAAT (Walters et al. 2015) with each primer modified at 5′ with the addition of Illumina pre-adapter and Nextera sequencing primer sequences.

Amplicons were generated using high-fidelity DNA polymerase (Q5 Taq; New England Biolabs). After an initial denaturation at 95°C for 2 min, the Polymerase Chain Reaction (PCR) conditions were as follows: 30 cycles of denaturation at 95°C for 15 s, annealing at 55°C for 30 s, and extension at 72°C for 30 s. A final extension step of 10 min at 72°C was performed. PCR products were purified using Merck MultiScreen PCR filter plates following manufacturer's instructions. MiSeq adapters and 8 nt dual-indexing barcode sequences (Kozich et al. 2013) were added during the second PCR amplification step. After an initial denaturation at 95°C for 2 min, the PCR conditions were eight cycles of denaturation at 95°C for 15 s, annealing at 55°C for 30 s, and extension at 72°C for 30 s, with a final extension of 10 min at 72°C.

The amplicon size was determined using an Agilent 2200 TapeStation system, and the library was normalized using the NGS Normalization Kit (Norgen Biotek), with subsequent quantification using the Qubit dsDNA HS kit (Thermo Fisher Scientific). The pooled libraries were further purified by gel extraction (QIAquick, Qiagen) and diluted to 400 pM with 7.5% Illumina PhiX. Denaturation of the library was achieved by adding a 10% final volume of 2N NaOH and incubating at room temperature for 5 min, followed by neutralization with an equal volume of 2N HCl. The library was then diluted to its load concentration using Illumina HT1 Buffer. The final denaturation was performed by heating to 96°C for 2 min, followed by cooling in crushed ice. The amplicon library was sequenced on an Illumina MiSeq using V3 600 cycle reagents.

### Bioinformatic analysis

Illumina demultiplexed sequences were processed in R using DADA2 (Callahan et al. 2016) to filter, denoise, and merge the sequences with the following parameters: primer sequences were removed with cutadapt (Martin 2011) and reads were truncated to 250 and 200 bases, forward and reverse, respectively. The filtering settings were as follows: maximum number of Ns (maxN) = 0 and maximum number of expected errors (maxEE) = (5,5). Sequences were dereplicated and the DADA2 core sequence variant inference algorithm was applied. Forward and reverse reads were then merged using the *mergePairs* function to produce amplicon sequence variants (ASVs). Chimeric sequences were removed using *removeBimeraDenovo* at default settings and sequence tables were constructed from the resultant ASVs. ASVs were subjected to taxonomic assignment using the *assignTaxonomy* function and SILVA v138.1 (Quast et al. 2013) training database.

### Bacterial community beta-diversity and environmental drivers

Examination of community composition was carried out in R using Microeco (Liu et al. 2020) and Vegan (Oksanen et al. 2015). Briefly, samples with <8000 reads were removed along with the removal of sequence variants identified as chloroplast and mitochondrial, or those not belonging to the domains of Bacteria or Archaea, and each sample's data were rarefied to 7555 reads before Bray–Curtis distance matrix calculations and non-metric multidimensional scaling (NMDS) analysis and plotting. Correlations between environmental variables were examined at the phylum level using the *cal_cor* function.

### Genomic trait assessment

To estimate the genome size of the identified taxa based on taxonomic information, ASVs were assigned values using representative genomes present in the IMG-ER public database (Markowitz et al. 2009). To do this, ASV taxonomies were matched iteratively to IMG-ER representative genomes as described by Markowitz et al. (2009). Genomic traits summarised by the database were downloaded (20th September 2024) for all isolate, single-cell amplified, and metagenome-assembled genomes. Based on the availability of taxonomies in the IMG-ER database, relevant genomic traits such as genome size, rRNA operon copy number, coding density, and GC content were retained. ASVs with unclassified genera were iteratively matched to higher taxonomic ranks. For example, if a particular genus did not have a genome size, the genome size of the family to which the genus was ascribed was used. To confirm rRNA copy numbers, a FASTA file containing the representative sequences of all ASVs was used to approximate rRNA copies using an Artificial Neural Network Approximator 16 (Miao et al. 2024). A consensus of the rRNA copies was used for subsequent analyses. The weighted mean rRNA copies, weighted mean GC, and weighted mean genome size of each sample were calculated using the rarified sequence abundance table.

### Social niche breadth

To determine the niche breadth of the identified ASVs within the dataset, we used the SNB metric, as described by Miejenfeldt et al. (2023). SNB quantifies the diversity of ecological interactions that a microbial taxon engages in by analysing co-occurrence patterns across samples. First, the taxonomic profiles were rarefied to equal sequencing depths to avoid bias from differential sequencing coverage. The rarefied data were then formatted and processed using the *calculate_SNB.py* script from the SNB analysis toolkit (https://github.com/MGXlab/social_niche_breadth_SNB). SNB scores for each ASV were calculated based on their co-occurrence with other taxa, reflecting the range of ecological networks an ASV interacts with, where higher scores indicate a more generalist strategy and lower scores reflect specialization. For each sample, a weighted mean SNB score was computed by combining the SNB scores of all ASVs present, weighted individually by their abundance (count), to provide an overall measure of the sample's community niche breadth. This approach was used to assess how environmental factors influence microbial generalization or specialization across samples.

### Phylogenetic and PhyloFactor analysis

For phylogenetic analysis, we generated a multiFasta file from the ASV table produced by the DADA2 pipeline. We utilized the DECIPHER package in R to align the DNA sequences. Following alignment, we constructed a Neighbor-Joining tree based on a distance matrix calculated from the aligned sequences using the maximum likelihood (ML) method, employing the general time reversible model for optimization. The resulting optimized ML tree served as the basis for subsequent phylogenetic analyses, including downstream PhyloFactor analysis (Washburne et al. 2017). This analysis incorporated taxonomic data, which were combined into a single column (lineage), along with the ASV abundances and associated metadata. To further evaluate potential biases in the estimated SNB across different lineages, including dominant and rare taxa, we assessed phylogenetic signals within the dataset. Specifically, we estimated Blomberg's K and Pagel's Lambda using phylogenetic generalized least squares regression for statistical inference (see Code availability).

As highlighted above, to analyse phylogenetic factors driving patterns in community composition, we employed the PhyloFactor package in R, where the input was the phyloseq object. We ensured that the phylogenetic tree was unrooted using the unroot function from the ape package, a critical step for the proper functioning of the PhyloFactor method. The ASV abundances were modelled as a response variable to the SNB score of the individual ASVs, applying a log-ratio transformation and establishing a significance threshold of 0.01. We specified several factors (*n* = 15) to be evaluated, from which the detailed summaries of the PhyloFactor analysis were extracted, selecting for significant taxa.

For data visualization, isometric log-ratio (ILR) boxplots, incorporating the ILR results into the phyloseq object through a custom function (see Code availability), were generated. This allowed for ILR stratification by high- or low-SNB scores, with separation based on median average weighted SNB scores. A phylogenetic tree was constructed to visualize the relationships between taxa and the SNB category, highlighting the significant taxa identified in the analysis (see Code availability).

### Data analysis and figures

R (v4.1) was used for data analysis and figure generation using the *ggplot2* package. The *mgcv* package was used for the regression analyses, while any differential analysis was performed using functions in base R.

## Results

### pH and OM together shape the microbial community composition

**Figure 2.**
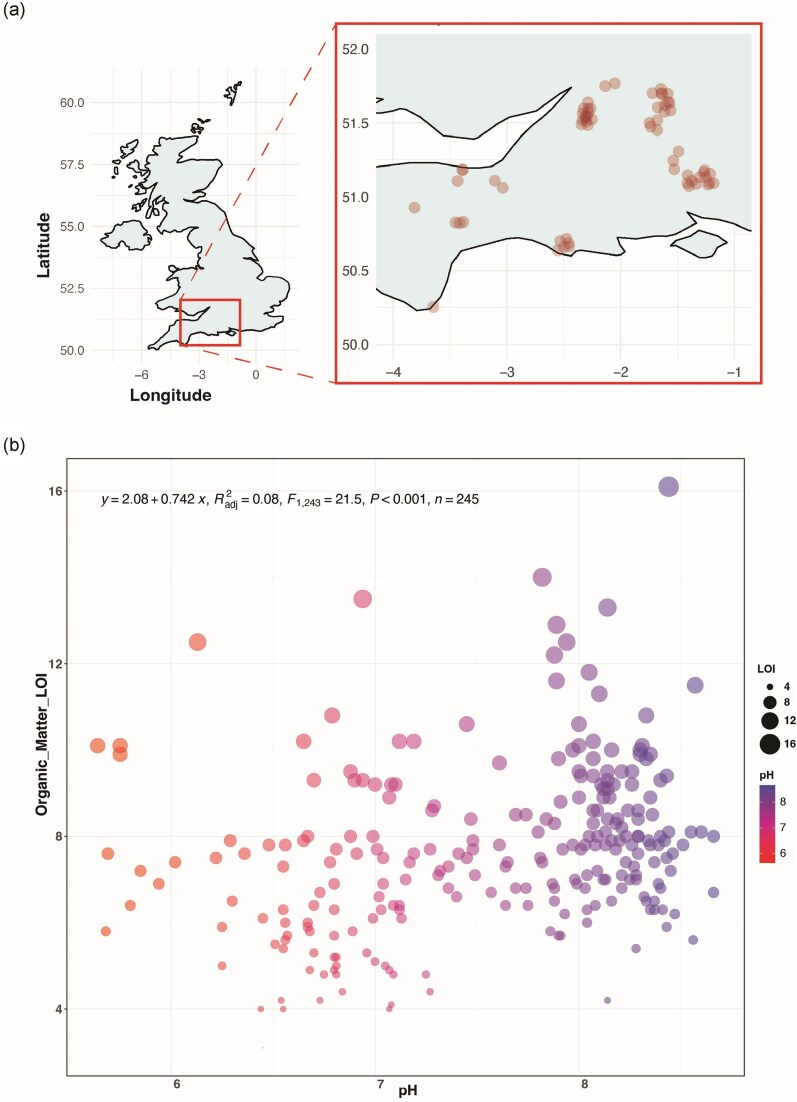
(a) Map of the United Kingdom showing the sample site distribution within southern England (*n* = 245; point alpha = 0.25); (b) relationship between soil samples’ OM content (LOI) and soil pH (*P* < .001, linear regression). The size of the filled circles indicates the measured OM of individual samples.

Agricultural fields have been shown to support the functioning of varied microbial communities. To understand how these intensively utilized soils vary in microbial composition, diversity, and abundance, we analysed 245 soil samples collected from arable, mixed arable-grass, and permanent grass fields across southern England (Fig. 2a).

**Figure 3.**
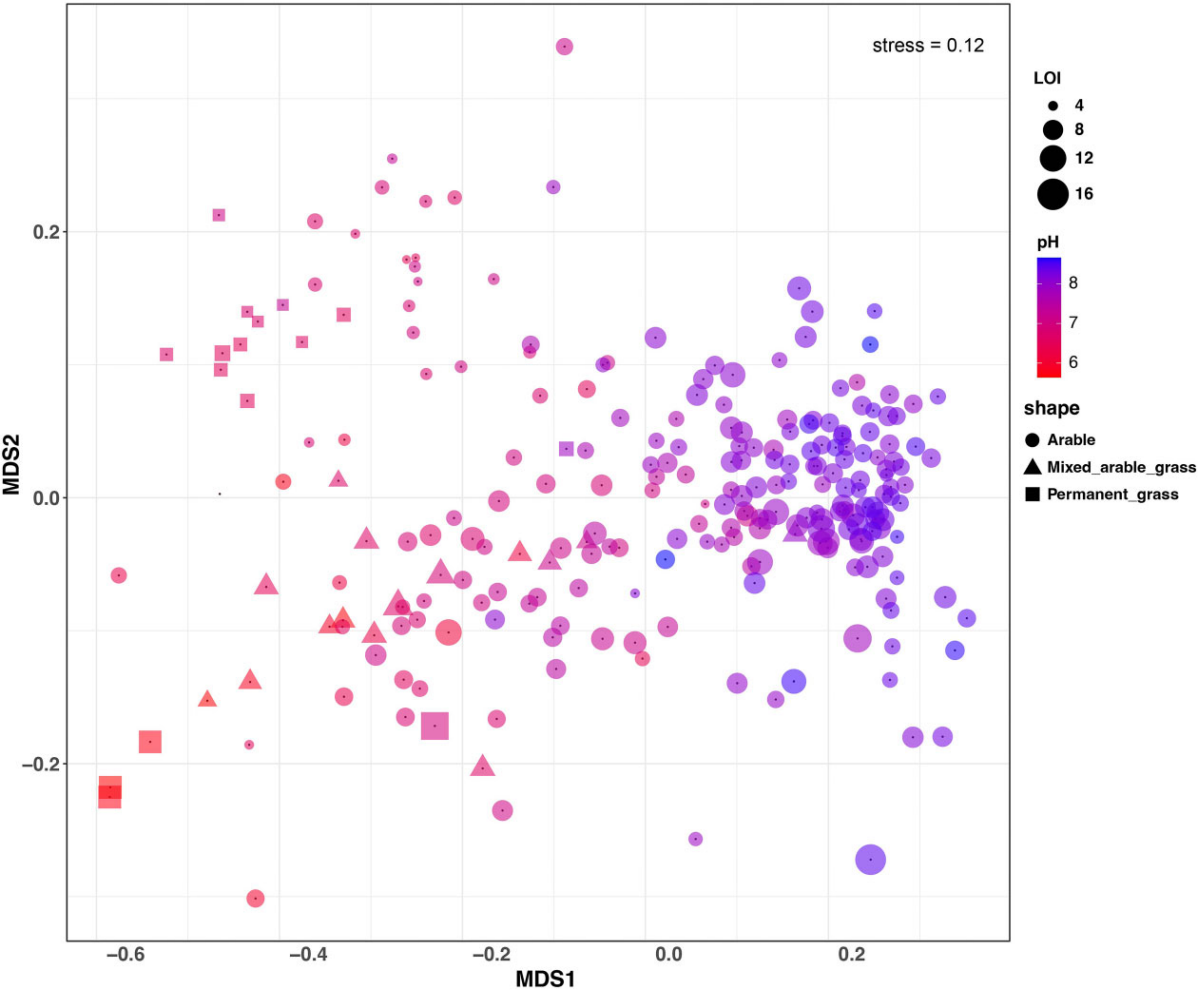
NMDS plot of soil beta diversity, where point size indicates the percentage of OM by LOI, point colour indicates soil pH, and shape indicates cropland rotation type. NMDS stress value (0.12) is indicated at the top right.

The average pH of these soil samples was 7.6 (range 5.6–8.7), and the average OM content (determined by LOI) was 7.7% (range 3.1%–16.1%). A weak but significantly positive relationship between pH and OM (*r*² = 0.08, *P* < .001) was observed (Fig. 2b). An ordination analysis (Fig. 3) revealed that the soil bacterial communities were primarily structured by pH. Furthermore, we observed a significant relationship between archaea: bacteria ratios within the samples and pH (Supplementary Fig. 4a and b).

### Broad taxonomic variability across arable and permanent grass fields

**Figure 4.**
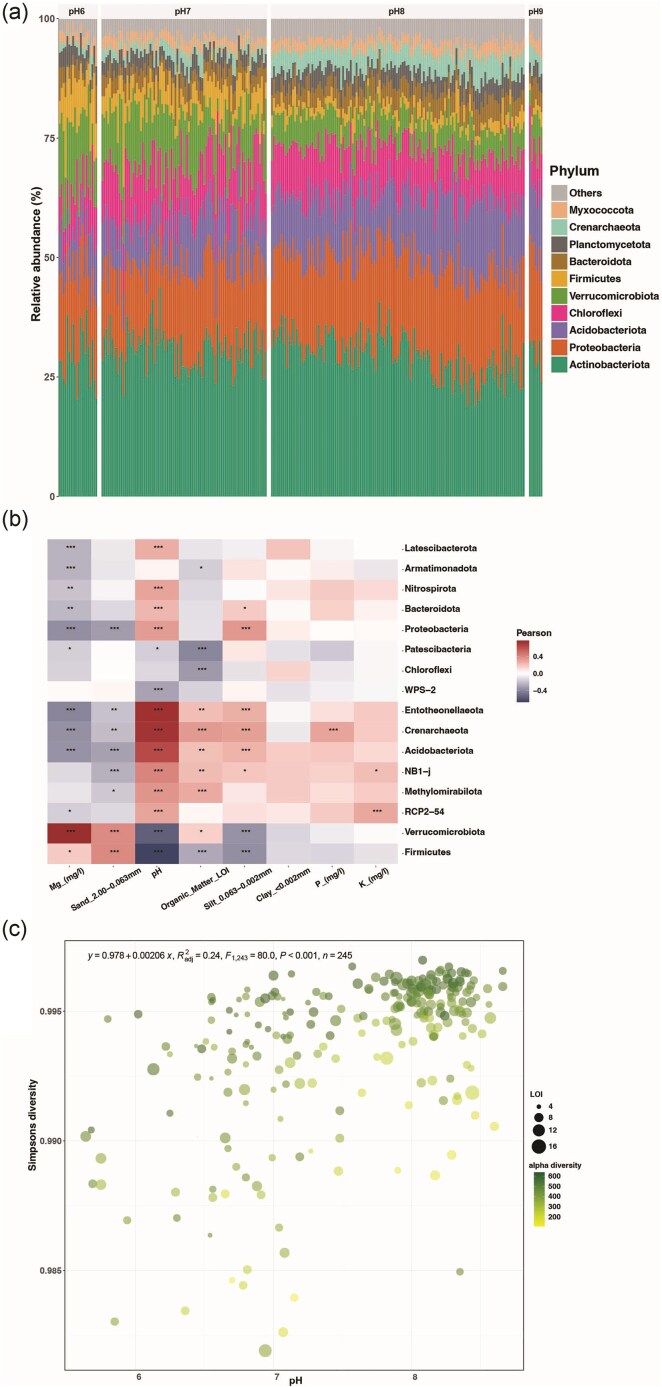
(a) Bar plot of the top 10 abundant phyla faceted by pH. pH6: samples ranging from pH 5.6–6.5, pH7: samples from pH 6.6–7.5, pH8: samples from pH 7.6–8.5, and pH9: samples from and above pH 8.5. (b) Correlations observed between all phyla and measured environmental variables (Spearman's correlation, *P*-values: *** .001, ** .01, * .05). (c) Relationship between Simpson's diversity index and soil pH (adj. *R*^2^ = 0.24, *P* < .001, linear regression), OM (LOI) as the point size, and the sample's alpha diversity as the colour gradient.

In-depth classification and analysis of the taxonomic components of the soils led to the observation of Actinobacteria, Proteobacteria, Chloroflexi, Verrucomicrobiota, Acidobacteria, Firmicutes, Bacteroidota, Crenarchaeota, Planctomycetota, and Myxococcota phyla being within the 10 most abundant and cosmopolitan phyla within the soils (Fig. 4a). Among these, the abundant phyla Entotheonellaeota, Crenarchaeota, Acidobacteria, Proteobacteria, Nitrospirota, NB1-j, Methylomirabilota, RCP2-54, and Bacteroidota were found to be positively correlated/associated with pH (Fig. 4b). A selection of the same taxa also positively correlated with OM. Conversely, taxa, such as Firmicutes, Verrucomicrobiota, and WPS-2, were negatively correlated with pH. Prokaryotic diversity (Simpson's) was found to increase with soil pH (Fig. 4c).

### Soil properties influence genomic traits of bacteria

**Figure 5.**
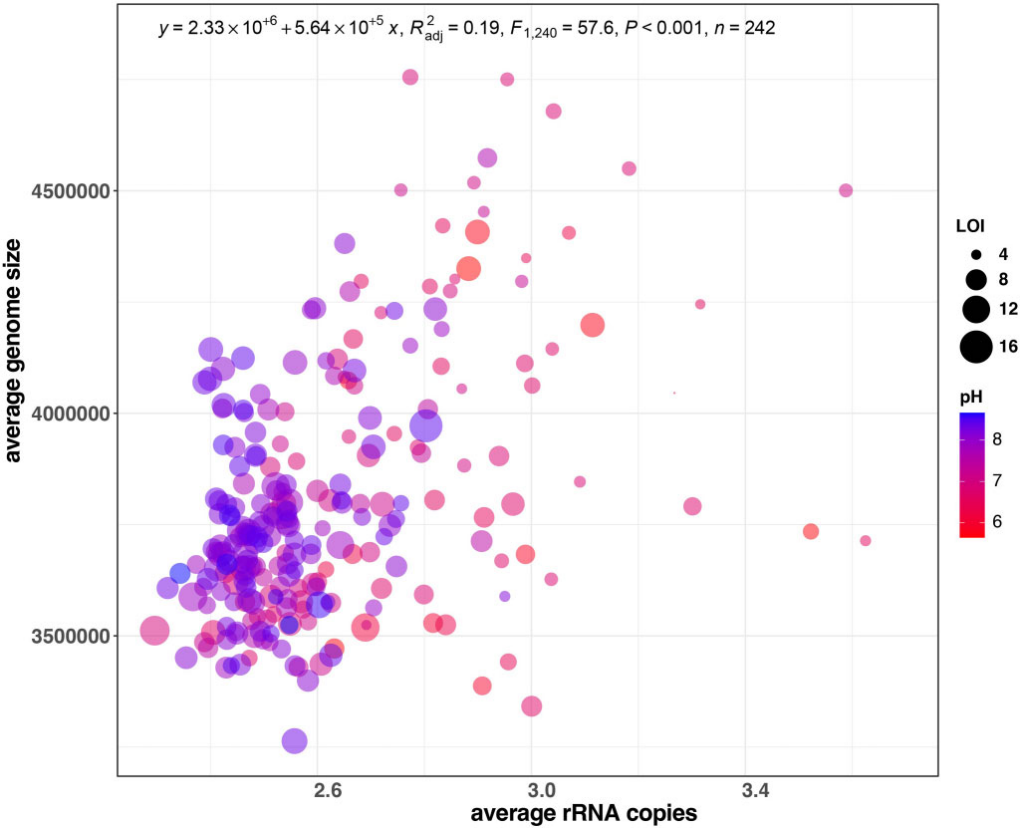
Scatter plot illustrating the significant positive relationship between the weighted mean genome size and weighted mean rRNA copy number, *R*^2^ = 0.19, *P* < .001, linear regression; OM (LOI) as point size and sample pH as colour gradient.

Given the observed variability in community composition and abundance, it is important to understand the effect of inherent soil properties on bacterial communities within these agricultural soils. To achieve this, we assessed the relationship between the dominant soil properties (pH and OM-LOI) and the genomic traits of bacteria found in these soils. Importantly, we focused on genome size and rRNA copy number which are indicative of life history, ecological strategies, and evolutionary adaptation. Genomic traits of the observed taxa were determined using genome representatives obtained from the publicly available IMG-ER database (see the 'Methods' section). We found a positive correlation between the weighted mean genome size and weighted mean rRNA copy number per genome (Fig. 5). Interestingly, we found that several samples with larger mean genome sizes and increased rRNA copy numbers were observed at decreasing pH levels. Generalized additive model analysis (Table 1) confirmed mean average rRNA copy number (ave.rrna) and mean average genome size (ave.gs) are significantly associated with soil pH (adj. *P* < .05; Table 2).

**Table 1.** Summary of generalized additive model—significant relationships of weighted mean rRNA copies (ave.rrna), weighted mean GC content (ave.gc), and weighted mean genome size (ave.gs), with soil pH.

Formula: pH ∼ s(Organic_Matter_LOI) + s(ave.gs) + s(ave.rrna) + s(ave.gc)					
Parametric coefficients:					
	Estimate	Std. Error	*t-*value	*P*r(>|*t*|)	
(Intercept)	7.56244	0.03526	214.5	<2e-16 ***	
Approximate significance of smooth terms:					
	Edf	Ref.df	*F*	*P*-value	Signif
s(ave.rrna)	1.906	2.399	52.175	< 2e-16	***
s(ave.gc)	2.994	3.815	5.033	0.000 924	***
s(ave.gs)	7.533	8.462	4.760	2.52e-05	***
s(Organic_Matter_LOI)	8.155	8.785	1.331	0.162284	

Signif. codes: 0 ‘***’ 0.001; ‘**’ 0.01; ‘*’ 0.05; ‘.’ 0.1; ‘ ’ 1.

R-sq.(adj) = 0.459; deviance explained = 50.5%.

GCV = 0.3303; scale est. = 0.30083; *n* = 242.

**Table 2.** Summary of generalized additive model—significant relationships of pH, OM (Organic_Matter_LOI), and weighted mean rRNA copy number (ave.rrna), with weighted mean SNB (ave.snb) score.

Formula: ave.snb ∼ s(Organic_Matter_LOI) + s(pH) + s(ave.gs) + s(ave.rrna) + s(ave.gc)					
Parametric coefficients:					
	Estimate	Std. Error	*t-*value	*P*r(>|*t*|)	
(Intercept)	0.566 325	0.0 005 157	1018	<2e-16 ***	
Approximate significance of smooth terms:					
	edf	Ref.df	*F*	*P*-value	Signif
s(pH)	7.869	8.663	74.332	< 2e-16	***
s(Organic_Matter_LOI)	1	1	11.912	0.000 668	***
s(ave.rrna)	6.771	7.866	5.98	1.53E-06	***
s(ave.gc)	2.884	3.682	2.937	0.032 546	*
s(ave.gs)	1	1	2.212	0.138 335	

Signif. codes: 0 ‘***’ 0.001; ‘**’ 0.01; ‘*’ 0.05; ‘.’ 0.1; ‘ ’ 1.

R-sq.(adj) = 0.865; deviance explained = 87.6%.

GCV = 7.0331e-05; scale est. = 6.4367e-05; *n* = 242.

### Bacterial niche breadth is influenced by soil properties

**Figure 6.**
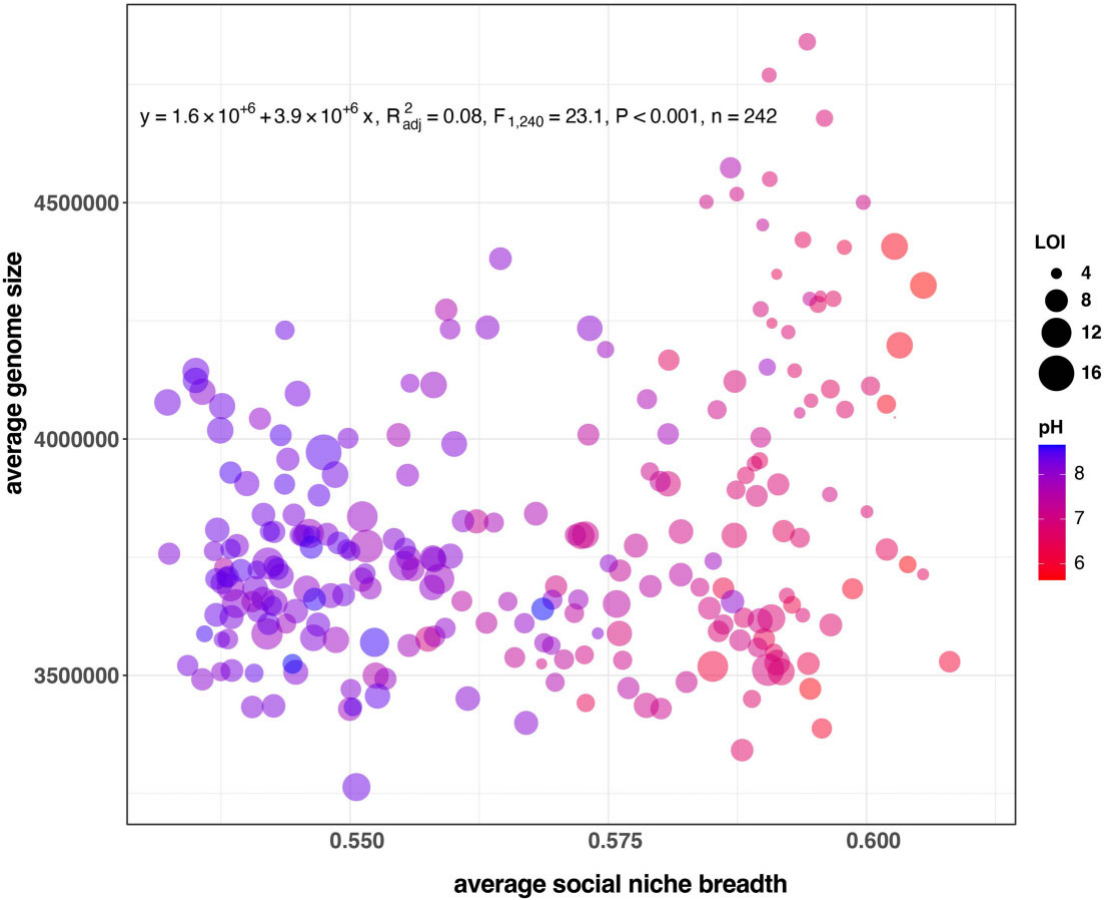
Scatter plot showing the positive relationship between the weighted mean genome size and weighted mean SNB, *R*^2^ = 0.08, *P* < .001, linear regression. The pH colour gradients showed an increasing mean SNB and mean genome size with acidity.

Since microbial composition and genomic traits were influenced by soil properties, we used the SNB of the individual taxa to calculate each sample's weighted mean SNB score. We found a clear distinction between the weighted mean SNB values, driven primarily by pH (Fig. 6). For example, at low pH levels, samples exhibited a higher SNB score, whereas the score was reduced at higher pH. The genome sizes within these communities varied and were diverse, with average genome sizes ranging from 3 263 535 bp to 4 840 393 bp (3 805 988 bp average). To rule out phylogenetic and abundance biases, we applied phylofactorization to assess phylogenetic signals linked to SNB scores. Of the 15 factors tested, 46% (7/15) were associated with high SNB, whereas the remainder were associated with low SNB (Supplementary Fig. 2). Notably, these SNB scores were independent of phylogenetic classification (Supplementary Fig. 3), suggesting that environmental factors primarily shape community composition. Factor 6 was representative of all factors where taxa across the bacterial tree were included in the low and high SNB clades along the tree (Supplementary Fig. 2). Additionally, phylogenetic and abundance factors did not bias SNB, as both dominant and rare taxa were represented in the low and high SNB groups (Supplementary Fig. 2). To further investigate the drivers of SNB, i.e., soil properties, we used generalized additive models to establish their importance and significance. Generalized additive model analysis confirmed that alongside pH and OM, both average GC content (ave.gc; Supplementary Fig. 1) and average rRNA copy number (ave.rrna) were significant drivers of SNB within the observed microbial community (adj. *P* < .05; Table 2).

## Discussion

Our study underscores the significant influence of pH and OM (measured as LOI) on shaping microbial community composition in soils from rotational arable (Aciego and Brookes 2008) and permanent grass fields, and demonstrates that the primary structuring factor for these microbial communities is pH. The association between soil pH and OM at the national scale and across all habitat types shows a clear relationship between acidity and increasing OM (LOI) (Emmett et al. 2010), demonstrating that this sample set does not conform to the broadly typical trend of high LOI at low pH (Kupka and Gruba 2022). This non-conformity can be explained due to the limited land use range, and because of the prevalence of samples with basic parent material where this trend breaks down (Armbruster et al. 2021). By focusing on highly managed soil systems situated on more basic parent material, we have constrained our study to soils that are under the most intensive forms of land use, where the conventional pH–LOI relationship is not consistently observed (Armbruster et al. 2021). This study examined the underlying microbial mechanisms and niche dynamics in these soils, which may differ significantly from soils under less intensive land use or management strategies, such as woodlands, heaths, and bogs.

Our taxonomic analysis of the soils revealed a broad diversity, spanning several phyla, including abundant soil bacteria (Delgado-Baquerizo et al. 2018, Bickel and Or 2021) such as Actinobacteria, Proteobacteria, Planctomycetota, Chloroflexota, and Verrucomicrobiota. A positive correlation between pH and OM with Entotheonellaeota, Acidobacteria and the archaea Crenarchaeota, suggests that these taxa may play critical roles in adapting to and mediating the effects of these environmental factors, providing further insight into the ecological strategies employed by these microbial communities. Indeed archaeal taxa have previously been identified as good indicators of soil OM content (Armbruster et al. 2021). Adaptions to low-pH environments or high OM levels may be due to specialized metabolic pathways or ecological interactions that are adventitious. Malik et al. (2017) previously reported taxa associated with low and high pH gradients with specific functional adaptations such as increased respiration and metabolism of aromatic compounds. Alternatively, microbial interactions, both mutual and antagonistic, may shape the composition and responses of the microbial community (Ratzke and Gore 2018). The role of soil carbon fluxes as a key function of microbial interactions (Wu et al. 2024) is further supported by Garcia et al. (2023) who reported that soil carbon levels are influenced by microbial interactions. Furthermore, Cole et al. (2024) recently reported that in low-pH soils, land use intensification increases microbial physiological constraints and decreases carbon use efficiency. These findings contribute to a growing body of knowledge on the complex interactions between soil properties and microbial community structure, particularly in managed ecosystems.

The observed variability in microbial community composition and abundance across different soil types further led us to hypothesise that microbial genomic traits may reflect adaptive strategies. The positive correlation between genome size and rRNA copy number across these soils suggests that these genomic traits may be key indicators of microbial life history strategies, ecological adaptability, and evolutionary processes. Interestingly, we found that larger genomes and increased rRNA copies were associated with lower pH (see Table 1), suggesting soil-specific adaptations. Our observations are in agreement with the recent report by Wang et al. (2023), where genome sizes across soils from pH 3.7 to 7.2 decreased with higher pH, and also align with the findings of Malik et al. (2017), where at higher pH, a higher GC content is observed, leading to reduced genome size (Almpanis et al. 2018). Our results suggest that non-acidophilic bacteria, typically observed in soil, do not follow the genome streamlining trends reported by Cortez et al. (2022). Larger genomes and higher rRNA copy numbers may provide a competitive advantage in nutrient-poor, complex or lower pH environments by enabling more efficient resource utilization and rapid responses to environmental changes and opportunities. A study by Wang et al. showed that soil pH is the key driver of microbial community composition in farmland soils compared to nutrients (Wang et al. 2019). However, given the effect of pH, on nutrient availability, it is conceivable that low pH soils may have higher nutrient availability, and therefore nutrient complexity, compared to higher pH soils (Neina 2019, Barrow and Hartemink 2023). Collectively, we hypothesise that this is likely because of lower pH soils being more environmentally and spatially variable. Although specific gene types are not associated with bacterial pH preferences (Ramoneda et al. 2023), anion/cation transporters, phosphatases, and efflux pumps have been identified in taxa that share pH associations (Ramoneda et al. 2023).

These results emphasize the importance of considering genomic traits when assessing microbial community dynamics in response to soil properties, as they offer valuable insights into the adaptive strategies of microbial taxa in different ecological contexts. Furthermore, mean sample SNB estimates revealed that pH was a major determinant of niche differentiation within the microbial community. It is likely that taxa in low (pH 5.5) to neutral pH soils are diverse (Luan et al. 2023) and may possess broader ecological niches, potentially due to the need to exploit a wider range of resources or tolerate more variable conditions. This finding supports the idea that metabolic versatility, which is closely correlated with genome size, is higher in communities inhabiting low pH soils. As previously stated, Wilhelm et al. (2023) demonstrated a positive relationship between genome size and rRNA copy number in soil bacteria; however, Wilhelm et al. (2023), our findings indicate that pH, rather than carbon content, is the dominant factor influencing genome size in the soils we examined. This suggests that care must be taken when using the average genome size as a proxy for carbon content or soil health, as pH may play a more significant role in shaping microbial genomic traits than previously thought. Additionally, Malik et al. (2018) identified a pH threshold of 6.2, above which significant shifts in carbon use efficiency occur, leading to smaller, more specialized genomes with narrower SNB at higher pH levels. The diversity in genome sizes coupled with SNB indicates that microbial taxa may employ different strategies to cope with the challenges posed by varying pH levels and significant additional drivers, such as genome size and rRNA copy number. Our results are in line with the hypothesis that larger genomes are associated with increased metabolic capacities (Piton et al. 2023), potentially explaining the increased niche breadth. Collectively, our findings emphasize the need to understand how soil properties influence microbial ecology, particularly in managed ecosystems, where soil conditions may deviate from broader, natural trends.

Overall, this study provides new insights into the relationships among soil physicochemical properties, microbial community composition, and genomic traits in arable and permanent grass soils. These findings challenge existing paradigms and highlight the need for further research on the specific mechanisms by which microbial communities adapt to and thrive in soils with atypical pH and OM relationships. By integrating taxonomic, genomic, and ecological analyses, this study offers a comprehensive understanding of the factors that drive microbial diversity and function in these important agricultural ecosystems.

## Supplementary Material

xtaf008_Supplemental_Files

## Data Availability

The code used for SNB analysis along with the R script used for figure generation with the associated files were uploaded to Zenodo. The files are publicly available and can be accessed via https://zenodo.org/records/13912858. Raw sequence files are available via the Sequence Read Archive under BioProject ID PRJNA1168686.
