# Supplementary material for: Soil properties in agricultural systems affect microbial genomic traits
Source: FEMS Microbes. 2025 Jun 24;6:xtaf008. doi: 10.1093/femsmc/xtaf008 (PMC12231137; doi:10.1093/femsmc/xtaf008)
Supplement: xtaf008_Supplemental_Files [file xtaf008_supplemental_files.zip › FEMSMC-2024-050.R1 one sentence summary.docx]

Bacterial genomic traits are related to soil properties in agricultural systems
