# Supplementary figures and images for: Soil properties in agricultural systems affect microbial genomic traits
Source: FEMS Microbes. 2025 Jun 24;6:xtaf008. doi: 10.1093/femsmc/xtaf008 (PMC12231137; doi:10.1093/femsmc/xtaf008)

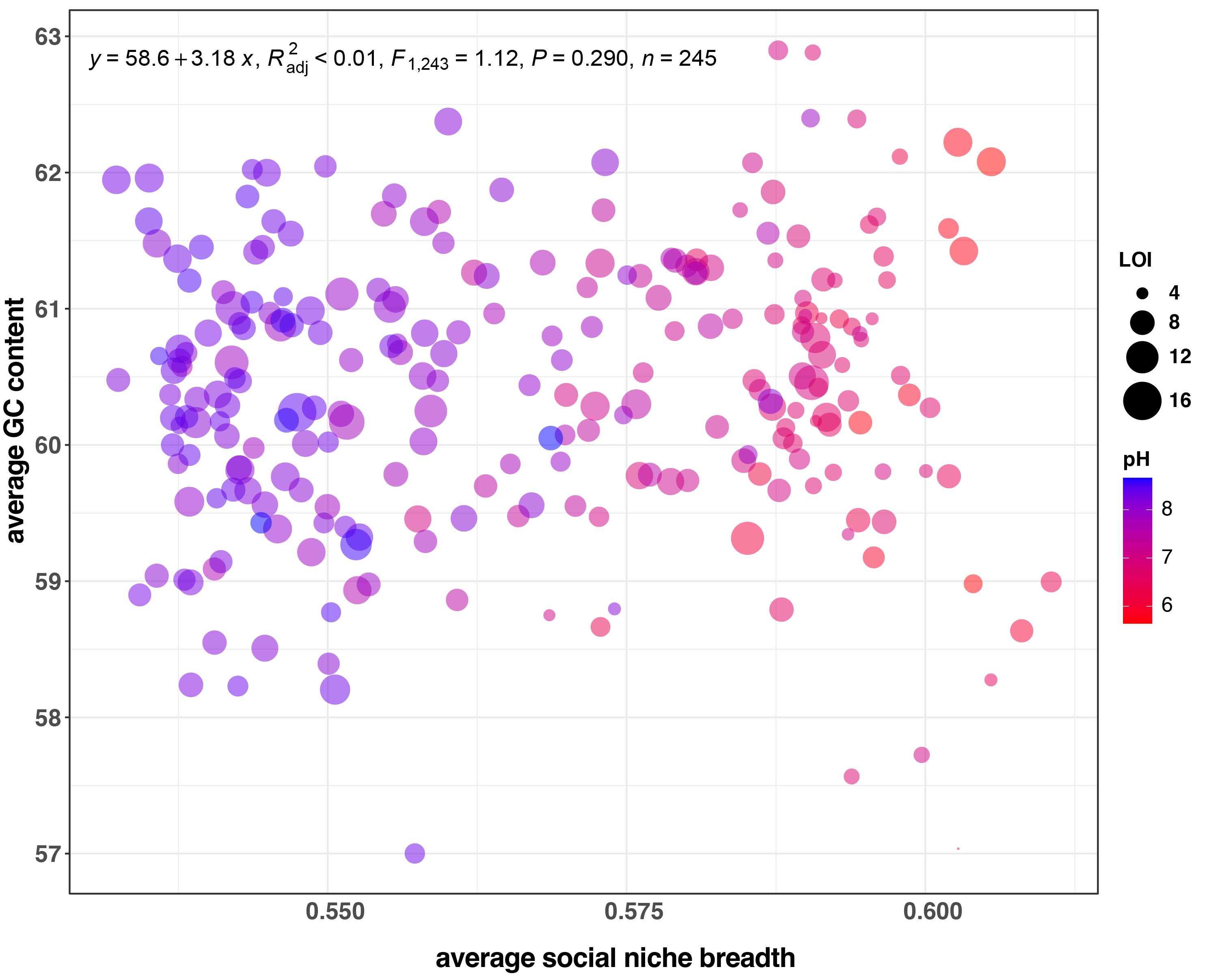

Supplement: xtaf008_Supplemental_Files [file xtaf008_supplemental_files.zip › suppfig1_GC_v_SNB.jpg]

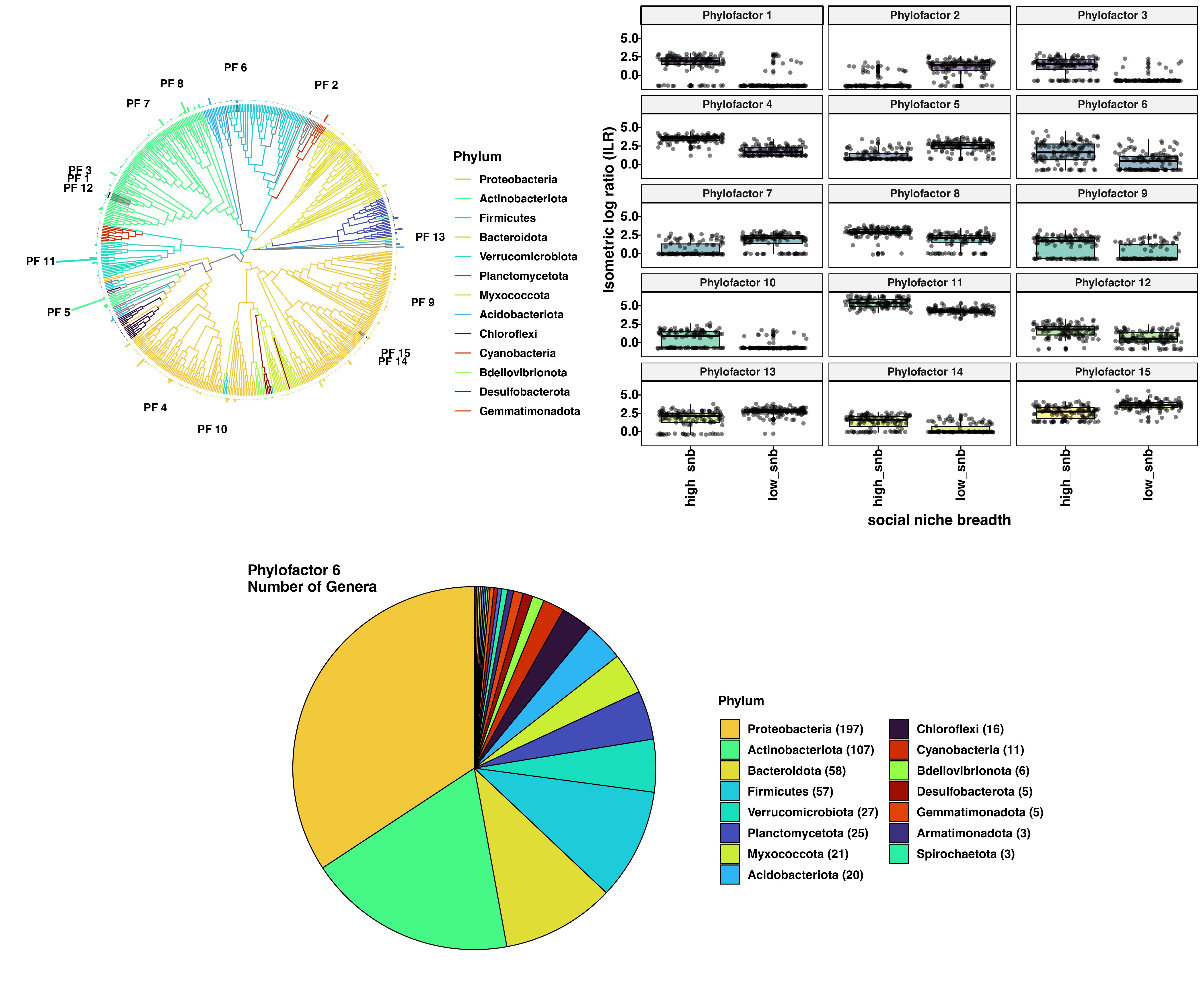

Supplement: xtaf008_Supplemental_Files [file xtaf008_supplemental_files.zip › suppfig2_pf_new_tree_boxplot_pie_allfactors.jpg]

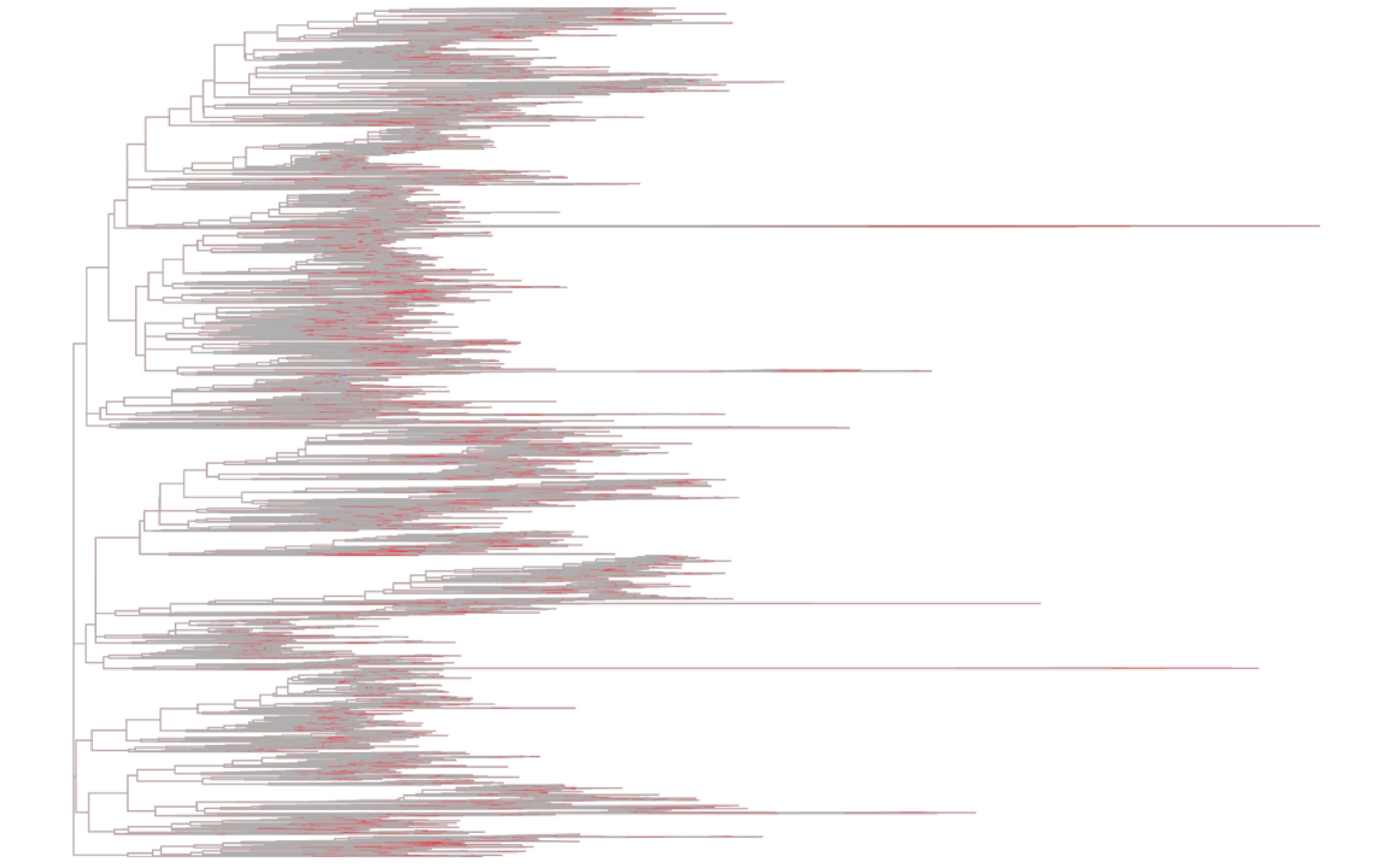

Supplement: xtaf008_Supplemental_Files [file xtaf008_supplemental_files.zip › suppfig3_tree.jpg]

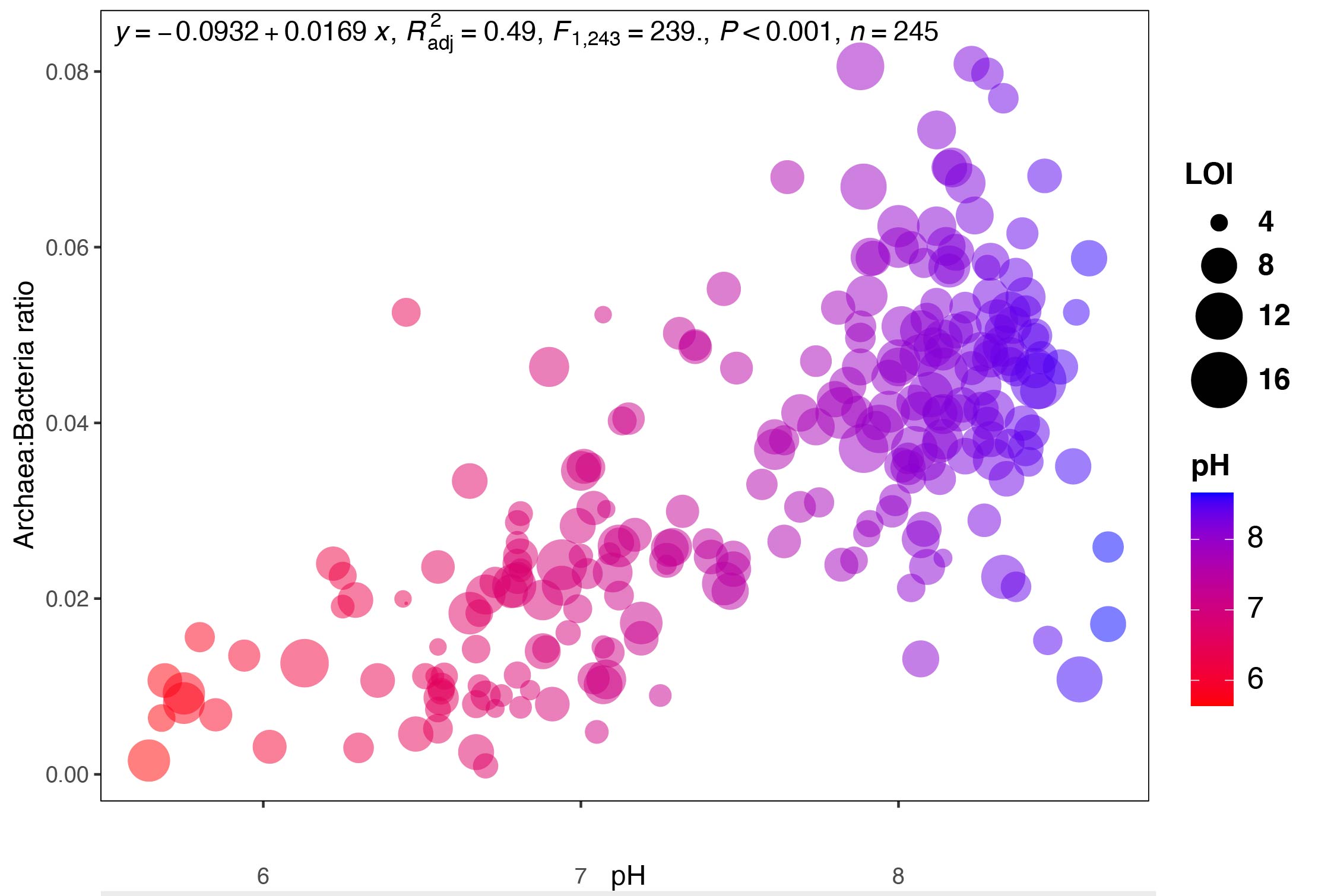

Supplement: xtaf008_Supplemental_Files [file xtaf008_supplemental_files.zip › suppfig4a_archaea_bacteria_ratio_scatter-01.jpg]

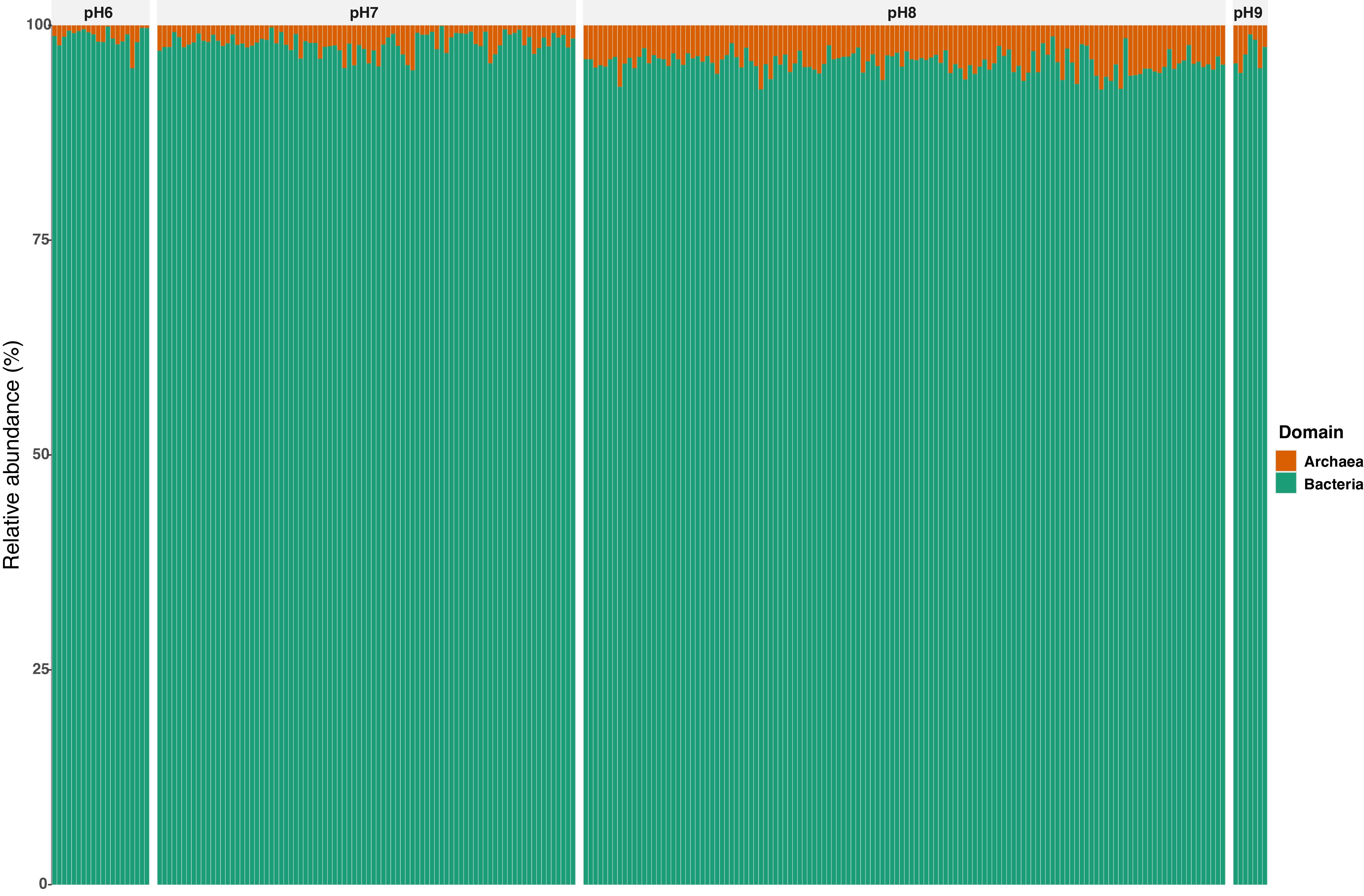

Supplement: xtaf008_Supplemental_Files [file xtaf008_supplemental_files.zip › suppfig4b_archaea_bacteria_ratio_barplot.jpg]
