# Supplementary material for: Soil properties in agricultural systems affect microbial genomic traits
Source: FEMS Microbes. 2025 Jun 24;6:xtaf008. doi: 10.1093/femsmc/xtaf008 (PMC12231137; doi:10.1093/femsmc/xtaf008)
Supplement: xtaf008_Supplemental_Files [file xtaf008_supplemental_files.zip › FEMSMC-2024-050.R1 supplementary data - reviews and author response.docx]

**Reviews of manuscript FEMSMC-2024-050**

**Reviewer 1**

**Recommendation: Minor Revision**

This paper examined the relationships between soil properties and bacterial traits within highly managed agricultural soil systems subjected to arable crop rotations or management as permanent pasture. They assessed bacterial communities and calculated amplicon trait scores and niche breadth trait scores of each amplicon. This manuscript is strong and well written, however it does have some minor changes that I suggest to improve the readibility and conhesiveness of the paper. The authors found that pH was the primary driver of bacterial traits. However, they do not indicate what the hypothesized mechanism is for this overarching response. In fact, they do not include hypotheses at all in the Introduction, and the paper could benefit from increased conceptual figures and diagrams that relate to articulated hypotheses. The methods were appropriate for addressing the aims of their study and the results and figures were aligned with their narrative. The Discussion was appropraite and situated their study well within the published literature. I recommend this manuscript for publication once they have address my concerns and revised the manuscript accordingly to elucidate hypotheses and provide a conceptual diagram of their research framework.

**Reviewer 2**

**Recommendation: Minor Revision**

The authors studied the relationship between microbial composition and physicochemical properties, in particular pH and organic matter, in arable, mixed arable / grassland, and permanent grassland soils in southern UK. They conducted an extensive survey of 245 samples from 68 fields, analyzing microbial composition using metabarcoding. The study showed that the pH had the greatest effect on microbial community, although organic matter also contributed. It is a nice, focused study.

Minor comments that should be addressed before publishing.

- Please add more detail on the land use for the samples. What type of agriculture – ie legume plants or grains? Was the grassland used for grazing, and if yes, which animals. The mixed arable/grassland – how recent was it switched or how often does it alternate.

- Fig 1A – needs clarification. The red dots are on top of each other, and some are light red while others are dark red. It is not clear that 68 areas were sampled from this image. I suggest zooming into the sample region and providing a clear detailed image. It is not necessary to show the entire country.

- The Results section blends results and discussion, primarily in the first 2 sub-sections of the results. I suggest merging the results and discussion, “Results and discussion” or removing the discussion components from the results and transferring to discussion.

- In the results (and discussion), the authors refer consistently to LOI, which is a method of measuring organic matter (OM) of the soil. It is easier to relate to and read if you refer to OM rather than the method used for measure. For example, the heading in results section (line 210) “pH and LOI together shape the microbial community composition”. It is not the LOI that shapes the community but rather the OM in the soil that contributes to shaping the community. I suggest to change this throughout the manuscript.

- All of the figures need larger text on the axes, listing phyla, and ***. Presently they are disproportionately small relative to the figure.

- The conclusion is that the pH is the key factor affecting the microbial traits. Could this be due to the increased release of nutrients at lower pH that contributes to the increased traits?

- The study identified Crenarchaeota, an Archaea commonly found in soil. Was there any difference between Bacteria and Archaea in their relationship within the soil of different land uses? Was it also affected by pH?

- line 213 – Awkward sentence. Remove …“in this study” … It is stated by the next part starting with "…we analyzed...".

- Line 233 – I think that should be Figure 3c (not figure 3b)?

**Associate Editor Recommendation: Minor Revision**

As you see below, the reviewers would like to see minor revisions to the manuscript to improve readability.

**EIC Decision: Minor Revision**

**Author's Response to Decision Letter for (FEMSMC-2024-050)**

Response to reviewer comments: FEMSMC-2024-050

Reviewer #1: This paper examined the relationships between soil properties and bacterial traits within highly managed agricultural soil systems subjected to arable crop rotations or management as permanent pasture. They assessed bacterial communities and calculated amplicon trait scores and niche breadth trait scores of each amplicon. This manuscript is strong and well written, however it does have some minor changes that I suggest to improve the readibility and conhesiveness of the paper.

Response: We thank the reviewer for recognising the effort and commending us on the manuscript. We appreciate their positive suggestions and have made the necessary changes to improve readability as outlined below.

Comment 1.1: The authors found that pH was the primary driver of bacterial traits. However, they do not indicate what the hypothesized mechanism is for this overarching response. In fact, they do not include hypotheses at all in the Introduction, and the paper could benefit from increased conceptual figures and diagrams that relate to articulated hypotheses.

Response: The reviewer makes a valid statement, and we apologise for the oversight on our part. We have now revised the manuscript in lines 73-81, where we highlight the possible hypothesis and overarching driver of our observations. We have also included a conceptual figure in the revised manuscript (see Figure 1).

Comment 1.2: The methods were appropriate for addressing the aims of their study and the results and figures were aligned with their narrative. The Discussion was appropraite and situated their study well within the published literature. I recommend this manuscript for publication once they have address my concerns and revised the manuscript accordingly to elucidate hypotheses and provide a conceptual diagram of their research framework.

Response: We thank the reviewer for this suggestion and have now included hypothesis statement(s) in lines 73-81. As noted above, we have also now included a conceptual diagram of our work, which is now listed as Figure 1 in the revised manuscript.

Reviewer #2: The authors studied the relationship between microbial composition and physicochemical properties, in particular pH and organic matter, in arable, mixed arable / grassland, and permanent grassland soils in southern UK. They conducted an extensive survey of 245 samples from 68 fields, analyzing microbial composition using metabarcoding. The study showed that the pH had the greatest effect on microbial community, although organic matter also contributed. It is a nice, focused study.

Response: We thank the reviewer for this comment and appreciate the positive feedback. Please see below responses to the specific comments.

Comment 2.1: - Please add more detail on the land use for the samples. What type of agriculture – ie legume plants or grains? Was the grassland used for grazing, and if yes, which animals. The mixed arable/grassland – how recent was it switched or how often does it alternate.

Response: We thank the reviewer for this comment and apologise for the oversight. The samples span a range of arable soils used for cereals (wheat and barley), alongside oil seed rape rotation crops. 16 samples among these were from permanent grasslands as well. We have included this information alongside detailed notes in the Methods section in lines 94-98, general details about the samples are available in supplementary table 1.

Comment 2.2: - Fig 1A – needs clarification. The red dots are on top of each other, and some are light red while others are dark red. It is not clear that 68 areas were sampled from this image. I suggest zooming into the sample region and providing a clear detailed image. It is not necessary to show the entire country.

Response: We agree with the reviewer and have replaced the figure with an updated version (Figure 2a) included a zoomed in section in the revised manuscript.

Comment 2.3: - The Results section blends results and discussion, primarily in the first 2 sub-sections of the results. I suggest merging the results and discussion, “Results and discussion” or removing the discussion components from the results and transferring to discussion.

Response: We thank the reviewer for this comment and apologise for the confusion. We have now edited the Results section to remove any discussion statements and thereby improve clarity. We have also updated the Discussion to reflect the changes in the revised manuscript.

Comment 2.4: - In the results (and discussion), the authors refer consistently to LOI, which is a method of measuring organic matter (OM) of the soil. It is easier to relate to and read if you refer to OM rather than the method used for measure. For example, the heading in results section (line 210) “pH and LOI together shape the microbial community composition”. It is not the LOI that shapes the community but rather the OM in the soil that contributes to shaping the community. I suggest to change this throughout the manuscript.

Response: We thank the reviewer for this suggestion and agree that it improves readability. We have now changed references to LOI throughout the revised manuscript to ‘organic matter’ measurements where appropriate.

Comment 2.5: - All of the figures need larger text on the axes, listing phyla, and ***. Presently they are disproportionately small relative to the figure.

Response: Thank you for the suggestion. We have now revised all the figures to improve clarity and readability.

Comment 2.6: - The conclusion is that the pH is the key factor affecting the microbial traits. Could this be due to the increased release of nutrients at lower pH that contributes to the increased traits?

Response: We thank the reviewer for this comment and suggestion to expand the discussion. We have now included this in our discussion in lines 322-326 in the revised manuscript.

Comment 2.7: - The study identified Crenarchaeota, an Archaea commonly found in soil. Was there any difference between Bacteria and Archaea in their relationship within the soil of different land uses? Was it also affected by pH?

Response: We thank the reviewer for the suggestion. While this was not in the original scope of the study, we chose to test the reviewer’s suggestion. Interestingly, there seems to be a positive correlation in the archaea:bacteria ratio, i.e. with increasing pH the ratio increases. We have now included this in the revised manuscript in lines 226-227 and as Supplementary figures 4 (a) and (b).

Comment 2.8: - line 213 – Awkward sentence. Remove …“in this study” … It is stated by the next part starting with "…we analyzed...".

Response: We thank the reviewer for this observation and have amended the paragraph within the manuscript, lines 218-221.

Comment 2.9: - Line 233 – I think that should be Figure 3c (not figure 3b)?

Response: We thank the reviewer for this observation and have amended the line within the manuscript, line 238.

**Reviews of manuscript FEMSMC-2024-050.r1**

**Associate Editor Recommendation: Accept**

The authors have addressed all the comments from the reviewers and incorporated them into the manuscript so that it reads very well. However I have several minor comments that should be addressed at some stage before publishing.

It is not grammatically correct to start a sentence with a number. Please revise the sentence starting on line 94.

Line 281 - refers to high LOI not high OM. This should conform to the rest of the text.

**EIC Decision: Accept**
